# P-Rex2, a Rac-guanine nucleotide exchange factor, is expressed selectively in ribbon synaptic terminals of the mouse retina

**DOI:** 10.1186/1471-2202-14-70

**Published:** 2013-07-11

**Authors:** David M Sherry, Bradley A Blackburn

**Affiliations:** 1 Department of Cell Biology, University of Oklahoma Health Sciences Center, 940 Stanton L Young Blvd, BMSB-553, Oklahoma City, OK 73104, USA; 2 Oklahoma Center for Neurosciences, University of Oklahoma Health Sciences Center, Oklahoma City, OK 73104, USA; 3 Department of Pharmaceutical Sciences, University of Oklahoma Health Sciences Center, Oklahoma City, OK 73104, USA

**Keywords:** Photoreceptor, Bipolar cell, Cytoskeleton, GEF, Rac GTPase, Actin

## Abstract

**Background:**

Phosphatidylinositol (3,4,5)-trisphosphate-dependent Rac Exchanger 2 (P-Rex2) is a guanine nucleotide exchange factor (GEF) that specifically activates Rac GTPases, important regulators of actin cytoskeleton remodeling. P-Rex2 is known to modulate cerebellar Purkinje cell architecture and function, but P-Rex2 expression and function elsewhere in the central nervous system is unclear. To better understand potential roles for P-Rex2 in neuronal cytoskeletal remodeling and function, we performed widefield and confocal microscopy of specimens double immunolabeled for P-Rex2 and cell- and synapse-specific markers in the mouse retina.

**Results:**

P-Rex2 was restricted to the plexiform layers of the retina and colocalized extensively with Vesicular Glutamate Transporter 1 (VGluT1), a specific marker for photoreceptor and bipolar cell terminals. Double labeling for P-Rex2 and peanut agglutinin, a cone terminal marker, confirmed that P-Rex2 was present in both rod and cone terminals. Double labeling with markers for specific bipolar cell types showed that P-Rex2 was present in the terminals of rod bipolar cells and multiple ON- and OFF-cone bipolar cell types. In contrast, P-Rex2 was not expressed in the processes or conventional synapses of amacrine or horizontal cells.

**Conclusions:**

P-Rex2 is associated specifically with the glutamatergic ribbon synaptic terminals of photoreceptors and bipolar cells that transmit visual signals vertically through the retina. The Rac-GEF function of P-Rex2 implies a specific role for P-Rex2 and Rac-GTPases in regulating the actin cytoskeleton in glutamatergic ribbon synaptic terminals of retinal photoreceptors and bipolar cells and appears to be ideally positioned to modulate the adaptive plasticity of these terminals.

## Background

The dynamic remodeling of the actin cytoskeleton is critical to the establishment of neuronal architecture and formation of synaptic connections, as well as structural and functional plasticity in response to normal or pathological stimuli. The Rho family of small GTPases, RhoA, Rac, and Cdc42, are key regulators of remodeling of the neuronal actin cytoskeleton and have distinct functions [reviewed in [1-3]]. Rho-family GTPases cycle between an inactive GDP-bound state and an active GTP-bound state, which is regulated by their interactions with a variety of guanine nucleotide exchange factors (GEFs) that promote activation by catalyzing the exchange of GDP for GTP, and GTPase activating proteins (GAPs) that promote inactivation by stimulating the GTPase to hydrolyze GTP to GDP. Therefore, the specific GEFs and GAPs expressed by a neuron and their localization within the cell determine the spatial and temporal activation of Rho-family GTPases and remodeling of the actin cytoskeleton.

Rac1 and Rac3 (also known as Rac1B) are Rho-family GTPases that are expressed in the central nervous system (CNS). Rac1 is important for outgrowth, guidance, and branching of neurites [2,3]. In contrast, activation of Rac3 appears to inhibit neurite outgrowth and may have a function antagonistic to that of Rac1 [4]. Several GEFs that specifically activate Rac GTPases have been identified and are known to affect development and structure of neuronal processes, consistent with the importance of actin cytoskeleton remodeling to neuronal structure and function (e.g., Tiam 1 and 2, Trio, Kalirin, P-Rex1 and 2) [5-14].

The Phosphatidylinositol (3,4,5)-trisphosphate-dependent Rac Exchanger family of proteins (P-Rex1 and P-Rex2) are GEFs that selectively activate Rac GTPases [15-17]. The P-Rexes are functionally unique in that they require simultaneous signals from PhosphoInositide-3-Kinase (PI3K) and G-protein coupled-receptor (GPCR) pathways for their activation. Thus, they integrate and communicate the coordinated signals in PI3K and GPCR pathways to the actin cytoskeleton through the activation of Rac GTPases [15,18,19].

Recent studies specifically implicate the P-Rex family of Rac-GEFs in the regulation of neurite growth and guidance, neuronal architecture, and functional plasticity. P-Rex1 is widely expressed by neurons in the developing central nervous system including neurons in the cerebral cortex and dorsal root ganglion [11]. P-Rex1 localizes to the leading edge of migrating cortical neurons and to neuritic growth cones [11,13]. Expression of a dominant negative form of P-Rex1 perturbs radial migration of cortical neurons [11], and knockdown of P-Rex1 in neuronally differentiated PC12 cells inhibits new neurite outgrowth while promoting the differentiation of existing neurites as indicated by enrichment of β tubulin [13]. Expression of P-Rex2 in the brain is much more limited and is most prominent in cerebellar Purkinje cells [12]. Knockout of P-Rex2 leads to aberrant dendritic architecture in cerebellar Purkinje cells and impaired cerebellum-mediated motor function [12]. Impaired P-Rex2 function also disrupts functional synaptic plasticity, interfering with late phase consolidation of long-term potentiation at the parallel fiber-Purkinje cell synapse [20].

Although it is clear that P-Rex function is important to cell migration, neurite differentiation and architecture during neural development, and to synaptic function in the adult brain, little is known about the distribution and function of P-Rex Rac-GEFs in specific synapses and circuits. To better understand the potential roles that P-Rex2 might play in regulation of the neuronal actin cytoskeleton in adult CNS neurons and circuits, we investigated the distribution of P-Rex2 in the mouse retina, a major model system for studies of neuronal and synaptic development and plasticity. To characterize the expression and localization of P-Rex2 in retinal neurons we analyzed immunolabeling for P-Rex2 in conjunction with well-known markers for specific retinal cell types and synapses at the light and confocal microscopic levels.

## Methods

### Animals and tissue preparation

Studies were performed using retinas from light-adapted adult wildtype C57BL/6 J mice (Jackson Laboratories, Bar Harbor, ME). Mice were kept on a 12-hour light: 12-hour dark cycle, with food and water available *ad libitum*. Animals were euthanized by rapid cervical dislocation. All animal procedures were approved by the University of Oklahoma Health Sciences Center Institutional Animal Care and Use Committee and conformed to the guidelines of the US Public Health Service and the Institute for Laboratory Animal Research.

Following euthanasia, eyes were enucleated, the cornea was punctured with a needle to facilitate fixation, and the eyes were immersion fixed in 4% paraformaldehyde in 0.1 M cacodylate or 0.1 M phosphate buffered saline (PBS) at pH 7.2 for 30 minutes to 2 hours at 4°C. After fixation, the anterior segment and lens were removed to create an eyecup. Eyecups were rinsed in PBS, cryoprotected in 30% sucrose in PBS, embedded in Optimal Cutting Temperature medium (OCT; Sakura Tissue Tek; VWR, West Chester, PA), and then rapidly frozen in liquid nitrogen. Frozen sections of 10–15 μm thickness were cut on a cryostat, collected on Superfrost Plus slides (Fisher Scientific, Pittsburgh, PA) and stored at −20°C to −35°C until use.

### Antisera, antibodies, and lectins

A panel of well-characterized antibodies and lectins directed against well known cell- and synapse-specific markers was used to localize P-Rex2 in the mouse retina. Details regarding source, immunogen, host species and dilutions for these reagents are provided in Table 1. Labeling patterns obtained in the current studies were consistent with previous reports and labeled appropriate cell types in the mouse retina.

**Table 1:** List of antibodies and lectins used for immunolabeling

**Antigen**	**Immunogen**	**Host**	**Dilution**	**Source**
Calbindin	Purified Calbindin from bovine kidney	mouse	1:300	Sigma, St Louis, MO (Catalog # C9848; clone CB-955)
GAD-65	Immunoaffinity purified GAD from rat brain	mouse	1:500	Developmental Studies Hybridoma Bank, U Iowa, (Catalog # GAD-6; clone GAD-6)
GlyT1	C-terminus peptide sequence from Rat GlyT1	goat	1:500	Chemicon, Temecula, CA (Catalog # AB1770)
MAP-1	Microtubules purified from rat brain	mouse	1:300	Sigma, St Louis, MO (Catalog # M4278; clone HM-1)
PKC	Human PKCα, aa. 270-427	mouse	1:100	B-D Transduction Labs, San Jose, CA (Catalog # 610107; clone 3/PKC)
PNA	— —	— —	1:20	Invitrogen-Molecular Probes, Carlsbad, CA (Catalog # L21409)
P-Rex2	Mouse P-Rex2, aa. 717-799	rabbit	1:1000	Dr. Heidi C.E. Welch, The Babraham Institute, Cambridge, England (affinity purified antiserum #78)
Synapto-tagmin 2	Zebrafish hindbrain membranes	mouse	1:200	Zebrafish International Resource Center, Eugene, OR (Catalog # znp-1; clone znp-1)
Syntaxin 1	Rat hippocampus synaptosomes	mouse	1:500	Sigma, St Louis, MO (Catalog # S0664; clone HPC-1)
VGluT1	Peptide, aa. 541–560 of rat VGluT1	guinea pig	1:1000–1:2000	Chemicon, Temecula, CA (Catalog # AB5905)
VGluT1	Peptide, aa. 493–560 of rat VGluT1	mouse	1:100–1:300	UC Davis/NIH NeuroMab Facility, Davis, CA (Catalog # 75–066; clone N28/9)

Mouse monoclonal anti-Calbindin was raised against purified bovine kidney Calbindin-D-28 K, and does not recognize other members of the EF hand calcium binding protein family. Immunolabeling patterns matched previous reports in the mouse retina [21]. Mouse monoclonal anti-glutamic acid decarboxylase, 65 kDa isoform (GAD-65) was raised against GAD purified from rat brain and recognizes a single band on western blots [22]. GAD-65 immunolabeling matched the pattern reported previously in mouse retina [21]. Goat polyclonal anti-GlyT1 was raised against a C-terminus peptide from rat GlyT1 and produced immunolabeling that matched previous reports in mouse retina [21,23]. Mouse monoclonal anti-MAP-1 was raised against microtubules purified from rat brain and recognizes a single band corresponding to MAP-1 on western blots of rat brain and does not cross react with MAP-2 [24]. MAP-1 immunolabeling of ganglion cell dendrites in the IPL matches previous reports [25]. Peanut agglutinin (PNA) is a lectin that recognizes β(1–3)-GalNAc carbohydrates and is a specific marker for the extracellular matrix surrounding cone outer segments and the base of cone terminals [26,27]. Mouse monoclonal anti-PKCα was raised against amino acids 270–427 of human PKCα, recognizes a single 82 kDa band on western blots of rat brain, and specifically immunolabeled rod bipolar cells in the mouse retina as appropriate [21,28,29]. Affinity purified rabbit polyclonal anti-P-Rex2 was raised against amino acids 717–799 of mouse P-Rex2 and recognizes a single band on western blots of mouse cerebellar lysates and does not cross-react with P-Rex1 [12]. Mouse monoclonal anti-synaptotagmin 2 was raised against adult zebrafish hindbrain membranes [30] and recognizes a single 60 kDa band in western blots of mouse cerebellar lysates and synaptosomes identified as synaptotagmin 2 by mass spectrometry [31]. Anti-synaptotagmin 2 labeled horizontal cells, Type 2 OFF-cone bipolar cells, and Type 6 ON-cone bipolar cells as previously reported for mouse retina [28,31]. Mouse monoclonal anti-syntaxin 1a was raised against rat hippocampal synaptosomes [32] and recognizes a single 35 kDa band on western blot corresponding to syntaxin 1a [33]. Immunolabeling matched patterns previously reported in retina and selectively labels amacrine cells and their processes and conventional synapses in the inner plexiform layer of the mammalian retina [23,32,34]. Two different antibodies directed against VGluT1 were used. The guinea pig polyclonal antibody and the mouse monoclonal antibody both recognize a single 52 kDa band on western blots and do not crossreact with VGluT2. Both VGluT1 antibodies produce immunolabeling of photoreceptor and bipolar cell terminals, matching the previously reported distribution of VGLUT1 in the retina [35,36].

### Immunolabeling

Methods for immunolabeling of paraffin and frozen sections were as described previously [23,37]. Paraffin sections were de-paraffinized, and then subjected to antigen retrieval through 100% methanol at −20°C for 20 min, followed by 1% NaBH_4_, then 0.1 M citrate buffer (pH 6.0) at 85-95°C for 45 min, after which they were exchanged to PBS or Hank’s buffered saline solution (HBSS, pH 7.4) and blocked and processed as described below for frozen sections. Frozen sections were thawed, rehydrated and treated with 1% NaBH_4_, rinsed, and exchanged to PBS or HBSS. Sections were then incubated in blocking solution (“blocker,” 10% normal goat serum + 5% bovine serum albumin + 1% fish gelatin + 0.5% triton X-100 in PBS or HBSS) for 2 hours at room temperature. Blocker was removed and primary antibody or a cocktail of primary antibodies for double or triple labeling experiments were applied for 1–2 days at 4°C. After removing primary antibody, sections were rinsed in PBS or HBSS, and fluorescent secondary antibody, or a cocktail of secondary antibodies ± PNA for multiple labeling experiments, was applied for 1 to 1.5 hours at room temperature. Sections were rinsed and coverslipped using Prolong Gold + DAPI (Invitrogen-Molecular Probes, Carlsbad, CA). Control sections processed in parallel using normal host sera substituted for primary antisera or by omitting primary antibody showed no labeling. Control sections that received only one primary antibody showed labeling in only the appropriate channel when treated with multiple fluorescent secondary antibodies.

### Imaging and analysis

Widefield visualization of immunolabeling was performed using an Olympus BX61WI fluorescence microscope (Olympus America, Center Valley, PA) equipped with a Hamamatsu ORCA-ER camera (Hamamatsu, Bridgewater, NJ). Slidebook software (Intelligent Imaging Innovations, Denver, CO) was used for image acquisition. Confocal microscopy was performed using an Olympus FluoView1000 confocal microscope (Olympus America, Center Valley, PA). Confocal images were acquired using a 60x oil immersion objective lens (NA = 1.42) and Olympus Fluoview software. To prevent bleed-through of signals between fluorescence channels, detector sensitivity and laser power were adjusted and images were collected sequentially in the different fluorescent channels. For preparation of figures, widefield or confocal images were exported to Photoshop (Adobe, Mountain View, CA) and brightness, contrast and threshold were adjusted to highlight specific labeling.

## Results

### P-Rex2 is expressed specifically in the synaptic layers of the retina

**Figure 1:**
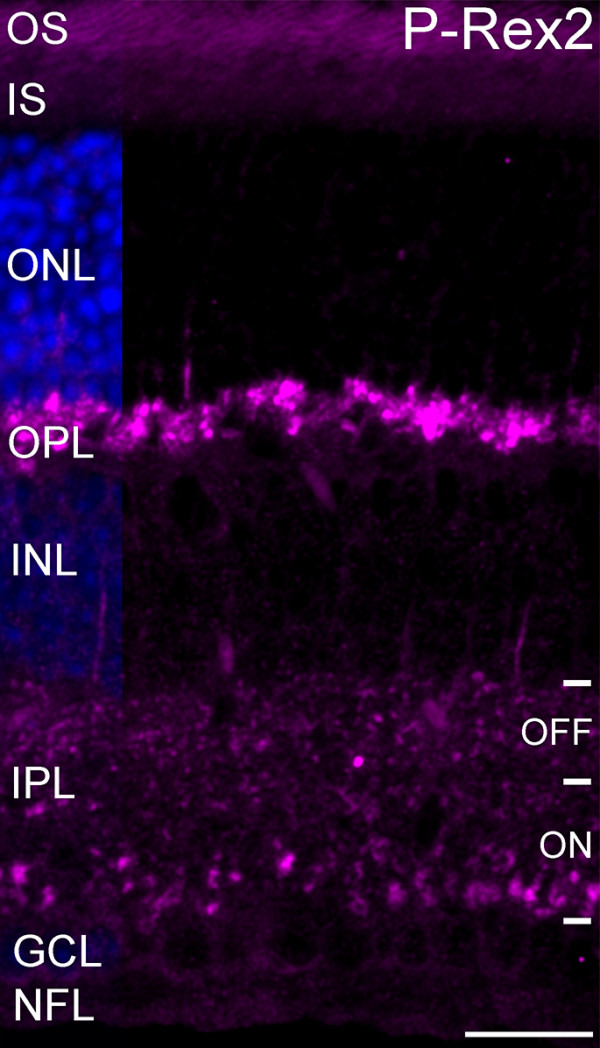
**P-Rex2 is localized to the synaptic layers of the retina.** Intense labeling for P-Rex2 is present in the OPL. Punctate labeling for P-Rex2 is distributed across the OFF and ON sublayers of the IPL. Weak labeling is present in some cell bodies in the position of bipolar cells in the INL. Labeling in the inner and outer segments of the photoreceptors is comparable to the autofluorescence commonly observed in these structures. Nuclei are counterstained with DAPI (blue) to illustrate nuclear layers. OS, outer segment; IS, inner segment; ONL, outer nuclear layer; OPL, outer plexiform layer; INL, inner nuclear layer; IPL, inner plexiform layer; GCL, ganglion cell layer. Scale bar = 20 μm.

Immunolabeling for P-Rex2 produced strong labeling in both the outer and inner plexiform layers (OPL and IPL, respectively) of the retina (Figure 1). Labeling in the OPL was intense and localized to a band of large terminals adjacent to the outer nuclear layer (ONL), consistent with labeling of photoreceptor terminals. Labeling in the IPL was characterized by large puncta distributed across the OFF and ON sublayers of the IPL, consistent with labeling of bipolar cell terminals. Some weaker diffuse labeling also was present in the IPL. Labeling in the inner and outer segments of the photoreceptors was comparable to the autofluorescence commonly observed in these structures.

### P-Rex2 is expressed specifically in rod and cone terminals in the OPL

**Figure 2:**
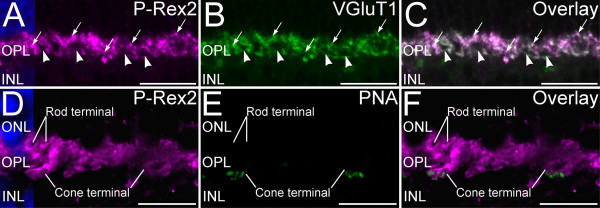
**P-Rex2 is present in rod and cone photoreceptor terminals. A**-**C**: P-Rex2 labeling in the OPL. **A**: P-Rex2 is expressed in numerous terminals in the distal portion of the OPL. **B**: VGluT1 is a specific marker for photoreceptor terminals. **C**: P-Rex2 colocalizes extensively with VGLUT1 in photoreceptor terminals (Overlay). Arrowheads indicate the large terminals of cones; arrows indicate smaller terminals of rods. **D**-**F**: Confocal imaging of P-Rex2 and peanut agglutinin (PNA) labeling in the OPL. P-Rex2 is expressed in the terminals of both rod and cone photoreceptors. **D**: P-Rex2 immunolabeling in photoreceptor terminals in the distal OPL. **E**: PNA specifically labels flat contacts made by OFF-cone bipolar cells at the base of cone terminals. **F**: Overlay of panels **D** and **E** demonstrates that P-Rex2 labeling is present in the large terminals of cones and smaller rod terminals. Maximum intensity projection of approximately 1.5 μm total thickness shown (5 optical planes at 0.3 μm step size). Nuclei are counterstained with DAPI (blue) in panels **A** and **D** to show the position of the ONL and INL. Confocal data are shown as maximum intensity projections of 1–5 optical planes acquired at 0.3 μm step size. ONL, outer nuclear layer; OPL, outer plexiform layer; INL, inner nuclear layer. Scale bars = 20 μm for **A**-**C**; 10 μm for **D**-**F**.

The characteristics of P-Rex2 labeling in the OPL suggested that P-Rex2 might localize specifically to photoreceptor terminals. Immunolabeling for P-Rex2 in combination with VGluT1, a specific marker for the glutamatergic terminals of rod and cone photoreceptors [35,36], showed extensive colocalization, confirming that P-Rex2 was present in photoreceptor terminals (Figure 2). To determine unequivocally whether P-Rex2 was present in the terminals of both rods and cones, we performed double labeling for P-Rex2 and peanut agglutinin (PNA), a marker that specifically labels synaptic contacts at the base of cone terminals [26,27]. Confocal microscopy showed that P-Rex2 labeling was present in the large terminals of cones identified by their PNA-positive synaptic contacts, as well as in the smaller and more numerous terminals of rods (Figure 2).

**Figure 3:**
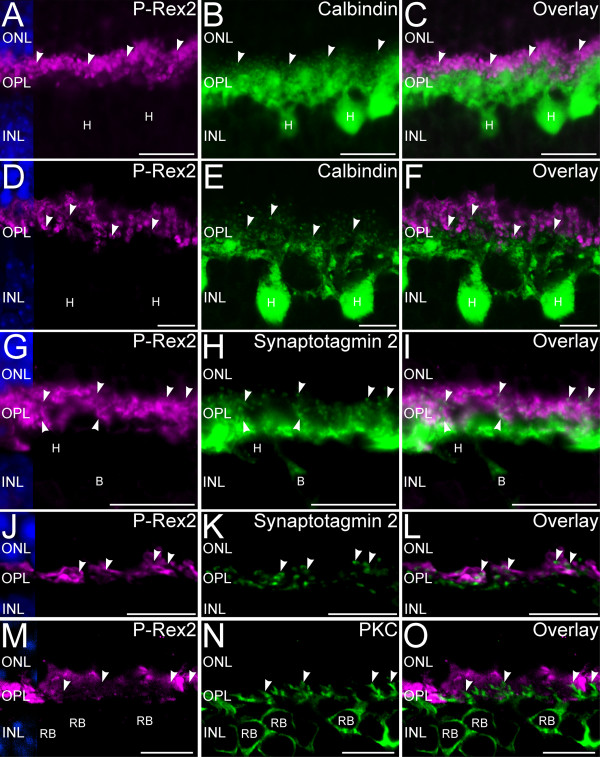
**P-Rex2 is absent in second-order neuronal processes in the OPL. A**-**C**: Widefield microscopy of P-Rex2 and calbindin labeling. **A**: P-Rex2 labeling in photoreceptor terminals. **B**: Calbindin labeling in horizontal cells **(H)** and their processes, including the fine processes that contact ribbon synapses (arrowheads). **C**: Overlay of **A** and **B**. **D**-**F**: Confocal imaging of P-Rex2 and calbindin labeling. **D**: P-Rex2 labeling in photoreceptor terminals. **E**: Calbindin is present in horizontal cell processes and their fine endings in photoreceptor terminals (arrowheads). **F**: Overlay of panels **D** and **E** confirms that horizontal processes do not contain P-Rex2. **G**-**I**: Widefield microscopy of P-Rex2 and Synaptotagmin 2 labeling. **G**: P-Rex2 is present in photoreceptor terminals. **H**: Synaptotagmin 2 is present in horizontal **(H)** and bipolar cells **(B)** and their processes, including the fine horizontal cell processes that contact photoreceptor terminals (arrowheads). **I**: Overlay of panels **G** and **H**. **J**-**L**: Confocal imaging of P-Rex2 and synaptotagmin 2 labeling. **J**: Photoreceptor terminals show P-Rex2. **K**: Synaptotagmin 2 is present in bipolar and horizontal cell processes, including the fine horizontal cell processes that contact photoreceptor terminals (arrowheads). **L**: Overlay of **J** and **K** confirms that P-Rex2 and synaptotagmin 2 do not colocalize. **M**-**O**: Confocal imaging of P-Rex2 and PKC labeling. **M**: P-Rex2 is present in photoreceptor terminals. N: PKC labeling in rod bipolar cells (RB) and their dendritescontacting rod terminals (arrowheads). **O**: Overlay of panels **M** and **N**. Rod bipolar cell dendrites do not show P-Rex2 labeling. DAPI (blue) shows nuclear layers in **A**, **D**, **G**, **J**, and **M**. Confocal images shown as single optical planes of approximately 0.3 μm thickness. ONL, outer nuclear layer; OPL, outer plexiform layer; INL, inner nuclear layer. Scale bars = 20 μm for **A**-**C** and **G**-**I**; 10 μm for **D**-**F** and **J**-**O**.

To assess whether P-Rex2 also was present in the processes of the second-order horizontal and bipolar cells in the OPL, we performed double labeling for P-Rex2 and markers that specifically label horizontal cell processes and the dendrites of bipolar cells (Figure 3). Double labeling for P-Rex2 and calbindin, a specific marker for horizontal cells and their processes [21], showed that P-Rex2 labeling was absent from the proximal portion of the OPL where the large processes of the horizontal cells ramify. Confocal microscopy confirmed that P-Rex2 also was absent from the fine processes of the horizontal cells that invaginate into the photoreceptor terminals to contact the photoreceptors at ribbon synapses. Double labeling for P-Rex2 and synaptotagmin 2, a specific marker for the processes of horizontal cells and Type 2 OFF-cone and Type 6 ON-cone bipolar cells [28,31], showed that the large processes of the second-order cells in the proximal portion of the OPL did not contain P-Rex2. Confocal microscopy demonstrated that P-Rex2 also was absent from the fine processes that contact the photoreceptor terminals. These fine processes from the second-order neurons occupied positions adjacent to the P-Rex2-positive photoreceptor terminals or positions within the invaginations in the photoreceptor terminals associated with the ribbon synaptic complexes. Similarly, rod bipolar cell dendrites, labeled for PKC [21], showed no labeling for P-Rex2. Together these results indicate that P-Rex2 in the OPL is specifically expressed in the ribbon synapse containing terminals of the rod and cone photoreceptors but not in the processes of second-order neurons.

### P-Rex2 expression in the IPL is restricted to bipolar cell terminals

**Figure 4:**
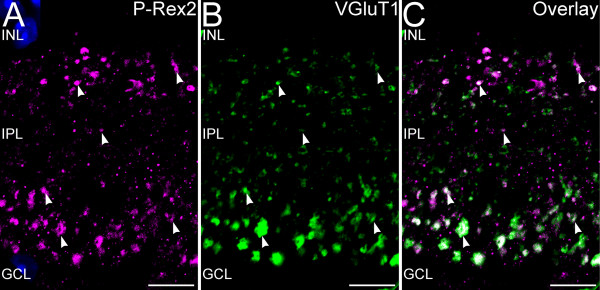
**P-Rex2 is localized to ribbon synaptic terminals of bipolar cells in the IPL.** Confocal imaging of P-Rex2 and VGluT1 labeling in the IPL (Single optical plane of approximately 0.3 μm thickness shown). **A**: P-Rex2 labeling is present in discrete puncta (arrowheads) located throughout the depth of the IPL. **B**: Labeling for VGluT1 identifies bipolar cell terminals throughout the IPL. The large terminals of rod bipolar cells along the inner edge of the IPL are prominently labeled. **C**: Labeling for P-Rex2 colocalizes extensively with VGluT1 labeling, indicating that P-Rex2 is localized to many bipolar cell terminals. Nuclei are counterstained with DAPI (blue) in panel **A** to illustrate the location of the INL and GCL. INL, inner nuclear layer; IPL, inner plexiform layer; GCL, ganglion cell layer. Scale bars = 20 μm.

The P-Rex2-positive terminals in the IPL showed the size and distribution characteristics of bipolar cell terminals. Labeling for P-Rex2 in combination with VGluT1, which labels the terminals of all bipolar cells [35,36], showed a high degree of correspondence (Figure 4). However, the correspondence of P-Rex2 and VGluT1 labeling was imperfect, with bipolar cell terminals that showed VGluT1 only and other small P-Rex2 puncta that did not show VGluT1 labeling. The intensity of P-Rex2 labeling also varied among bipolar cell terminals suggesting heterogeneity in P-Rex2 expression levels among bipolar cell terminals.

**Figure 5:**
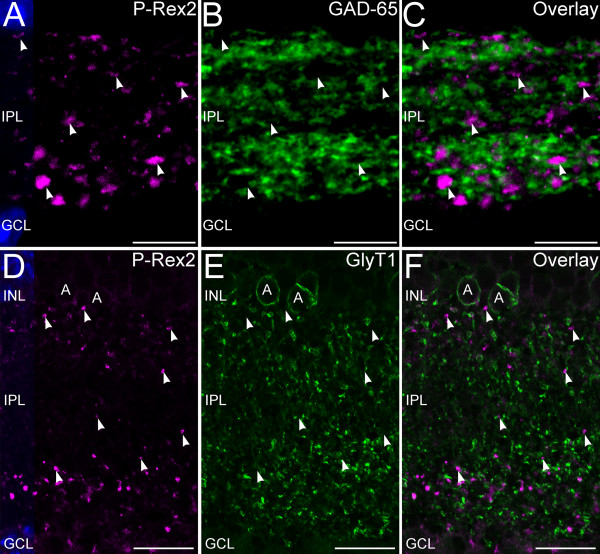
**Amacrine cells express little, if any, P-Rex2 in their processes and terminals. A**-**C**: Confocal imaging of P-Rex2 and glutamic acid decarboxylase 65 kDa (GAD-65) labeling in the IPL. **A**: P-Rex2 labeling is present in discrete puncta located at all levels of the IPL (arrowheads). **B**: Labeling for GAD-65, a marker for GABAergic amacrine cells, labels numerous synapses and processes throughout the IPL. **C**: Overlay of panels **A** and **B** shows that P-Rex2 and GAD-65 are independently distributed. **D**-**F**: Confocal imaging of P-Rex2 and Glycine Transporter 1 (GlyT1) labeling in the IPL. **D**: Terminals at all levels of the IPL (arrowheads) show labeling for P-Rex2. **E**: Labeling for GlyT1 is present in glycinergic amacrine cell bodies **(A)** and processes throughout the IPL. **F**: Overlay of panels **D** and **E** confirms that P-Rex2 and GlyT1 show independent distributions. Single optical planes of approximately 0.3 μm thickness shown. Nuclei are counterstained with DAPI in panels **A** and **D** to illustrate the location of the INL and GCL. INL, inner nuclear layer; IPL, inner plexiform layer; GCL, ganglion cell layer. Scale bars = 10 μm for **A**-**C**; 20 μm for **D**-**F**.

**Figure 6:**
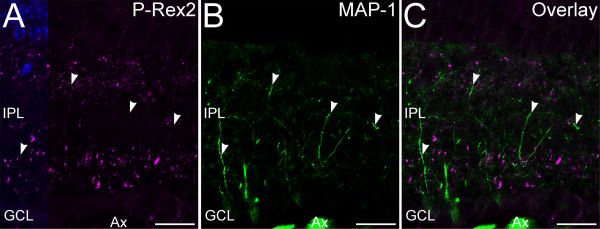
**Ganglion cell dendrites express little, if any, P-Rex2. A**-**C**: Confocal imaging of P-Rex2 and Microtubule-Associated Protein-1 (MAP-1) labeling in the IPL. **A**: P-Rex2 labeling is present in discrete puncta located throughout the IPL. **B**: Labeling for MAP-1 is present in ganglion cell dendrites throughout the IPL, as well as ganglion cell bodies in the GCL and their axons (Ax). **C**: Overlay of panels **A** and **B** shows that P-Rex2 and MAP-1 are independently distributed. Nuclei are counterstained with DAPI in panel A to illustrate the location of the INL and GCL. Single optical plane of approximately 0.3 μm thickness shown. IPL, inner plexiform layer; GCL, ganglion cell layer. Scale bars = 20 μm.

To test whether P-Rex2 also might be expressed in the conventional synapses of amacrine cells, we performed double immunolabeling for P-Rex2 and amacrine cell markers (Figure 5). Double labeling for P-Rex2 and the 65 kDa form of glutamic acid decarboxylase (GAD-65), a specific marker for GABAergic amacrine cells [21], revealed little evidence of colocalization of labeling for P-Rex2 and GAD-65, indicating that GABAergic amacrine cells express little, if any, P-Rex2. Similarly, there was little evidence of colocalization between P-Rex2 and Glycine Transporter 1 (GlyT1), a specific marker for glycinergic amacrine cells [21] or syntaxin 1a (not shown), a transmitter-independent marker for the synapses and processes of amacrine cells in the IPL [23,32,34]. To test whether P-Rex2 might be present in ganglion cell dendrites, we performed double labeling for P-Rex2 and Microtubule-Associated Protein 1 (MAP-1), a ganglion cell marker [25]. There was little if any colocalization of P-Rex2 and MAP-1 labeling (Figure 6).

Together these results indicate that P-Rex2 is localized specifically to the synaptic terminals of bipolar cells in the IPL, but is not present in the synapses or process of amacrine or ganglion cells.

### Identification of bipolar cell types expressing P-Rex2

To identify specific types of bipolar cells expressing P-Rex2 in their terminals, we performed double labeling for P-Rex2 in combination with markers for the terminals of specific rod- and cone-driven bipolar cell subtypes.

**Figure 7:**
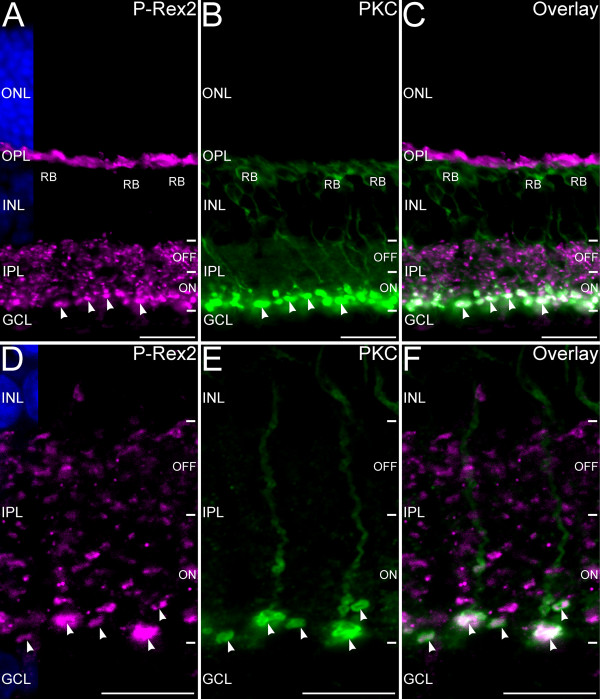
**P-Rex2 is expressed in rod bipolar cell terminals. A**-**C**: Widefield microscopy of double labeling for P-Rex2 and Protein kinase C (PKC), a rod bipolar cell marker. **A**: P-Rex2 is present in photoreceptor terminals in the OPL and in numerous bipolar cell terminals in the IPL. **B**: Labeling for PKC identifies rod bipolar cells (RB) and their terminals in the innermost portion of the ON sublamina of the IPL (arrowheads). **C**: The overlay of panels **A** and **B** shows that P-Rex 2 is localized to rod bipolar cell terminals in the IPL. Rod bipolar cell dendrites in the OPL did not show P-Rex2 labeling. **D**-**F**: Confocal imaging of P-Rex2 and PKC labeling in the IPL confirms that P-Rex2 (panel **D**) is localized to rod bipolar cell terminals (arrowheads) labeled for PKC (Panel **E**). There is little labeling for P-Rex2 in the axons of the rod bipolar cells coursing through the IPL. Panel **F** shows the overlay of panels **D** and **E**. Single optical plane of approximately 0.3 μm thickness shown. Nuclei are counterstained with DAPI (blue) in panels **A** and **D** to illustrate the locations of the nuclear layers. ONL, outer nuclear layer; OPL, outer plexiform layer; INL, inner nuclear layer; IPL, inner plexiform layer; GCL, ganglion cell layer. Scale bars = 20 μm for **A**-**C**; 10 μm for **D**-**F**.

The large P-Rex2-positive terminals in the innermost portion of the IPL showed the large size and placement characteristic of rod bipolar cells, which comprise a single cell type that can be identified by labeling for PKC [21]. Double labeling for P-Rex2 and PKC confirmed that rod bipolar cell terminals showed strong labeling for P-Rex2 (Figure 7). Interestingly, the distribution of P-Rex2 and PKC within a given terminal was not identical, with P-Rex2 labeling showing a more restricted spatial distribution than PKC labeling, indicating that P-Rex2 localized to specific domains within the terminal.

**Figure 8:**
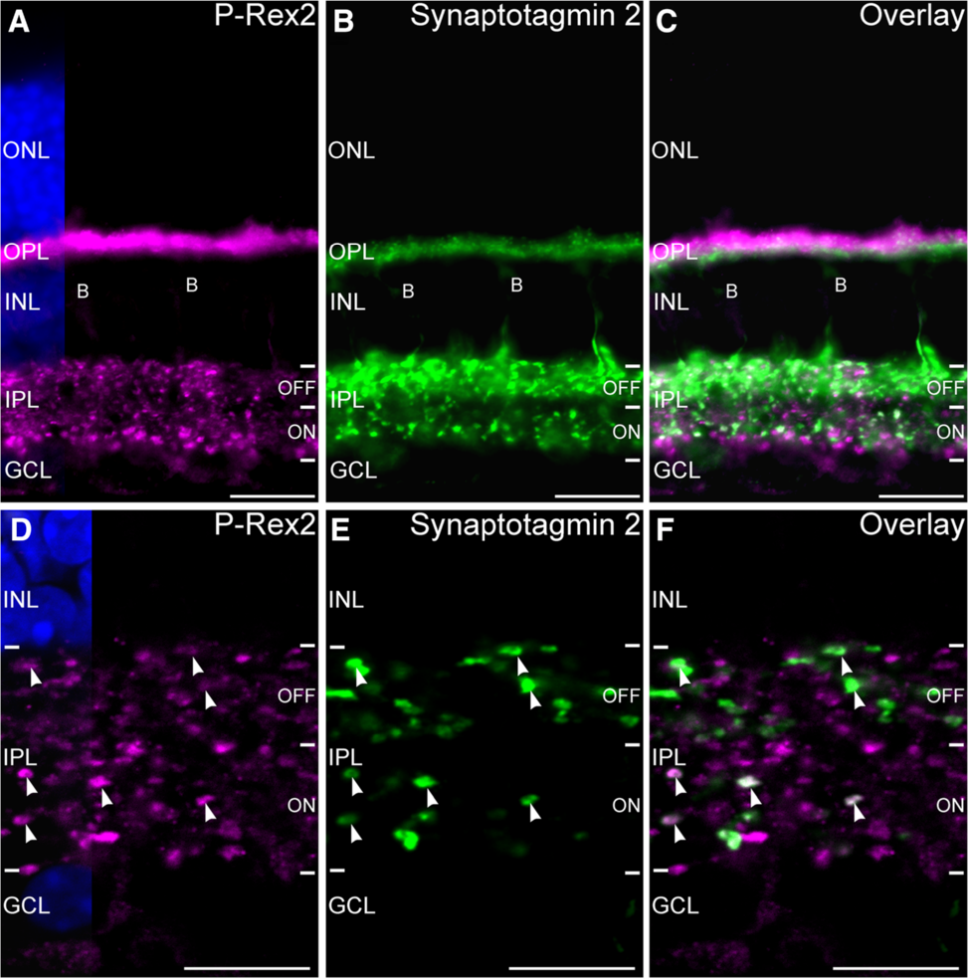
**P-Rex2 is expressed in the terminals of OFF- and ON-cone bipolar cells. A**-**C**: Widefield microscopy of double labeling for P-Rex2 and Synaptotagmin 2, a marker for Type 2 OFF-cone and Type 6 ON-cone bipolar cell terminals. **A**: P-Rex2 is present in photoreceptor terminals in the OPL and in numerous bipolar cell terminals in the ON and OFF sublaminae of the IPL. **B**: Labeling for Synaptotagmin 2 reveals an extensive plexus of Type 2 OFF-cone bipolar cell terminals in the OFF sublamina of the IPL. Type 6 ON-cone bipolar cell terminals form a sparser plexus in the ON sublamina of the IPL. Labeling of horizontal and bipolar cell processes is present in the OPL and faint labeling of Type 2 bipolar cell bodies **(B)** is visible in the INL. **C**: Overlay of panels **A** and **B** shows apparent colocalization of P-Rex 2 and synaptotagmin 2 labeling in the OFF and ON sublayers of the IPL. **D**-**F**: Confocal imaging of P-Rex2 and synaptotagmin 2 labeling in the IPL confirms that P-Rex2 (panel **D**) and synaptotagmin 2 (panel **E**) colocalize in the terminals of Type 2 OFF-cone and Type 6 ON-cone bipolar cells (arrowheads) in the OFF and ON sublayers of the IPL, respectively. Panel **F** shows the overlay of panels **D** and **E**. Intensity of P-Rex2 labeling varies considerably among individual Type 2 and Type 6 bipolar cell terminals, with some terminals showing no P-Rex2 labeling. Single optical plane of approximately 0.3 μm thickness shown. Nuclei are counterstained with DAPI in panels **A** and **D** to illustrate the locations of the nuclear layers. ONL, outer nuclear layer; OPL, outer plexiform layer; INL, inner nuclear layer; IPL, inner plexiform layer; GCL, ganglion cell layer. Scale bars = 20 μm for **A**-**C**; 10 μm for **D**-**F**.

Cone bipolar cells in the mouse retina comprise approximately 9 different types that terminate at characteristic depths in the IPL corresponding to their depolarizing (ON) or hyperpolarizing (OFF) light-driven responses [31,38]. The presence of P-Rex2-positive bipolar cell terminals throughout the depth of the IPL (see Figure 4) indicated that both ON- and OFF-cone bipolar cell subtypes expressed P-Rex2 in their terminals. To begin identifying specific cone bipolar cell types that contained P-Rex2 in their terminals, we performed double labeling for P-Rex2 and synaptotagmin 2, a specific marker for Type 2 cone bipolar cells (an OFF type) and Type 6 cone bipolar cells (an ON type) and their terminals in the IPL (Figure 8). There was extensive colocalization of P-Rex2 and synaptotagmin 2 labeling in the terminals of Type 2 cone bipolar cell terminals in the distal (OFF) sublayer of the IPL. However, some Type 2 cone bipolar cell terminals showed no P-Rex2 labeling, indicating that P-Rex2 levels can be heterogeneous among bipolar cell terminals of the same cell type. Similarly, there was extensive colocalization of P-Rex2 and synaptotagmin 2 labeling in the proximal (ON) sublayer of the IPL, indicating that the terminals of Type 6 cone bipolar cells also contained P-Rex2. Heterogeneous levels of P-Rex2 expression also were noted among Type 6 cone bipolar cell terminals. In addition, many other bipolar cell terminals throughout the OFF and ON sublaminae of the IPL showed single labeling for only P-Rex2, indicating that cone bipolar cell types in addition to the Type 2 and Type 6 cells also expressed P-Rex2 in their terminals.

## Discussion

These studies establish that P-Rex2, a GEF that specifically activates Rac small GTPases, is expressed in a cell- and synapse-specific manner in the retina. P-Rex2 localized to the synaptic terminals of photoreceptor and bipolar cells, as indicated by extensive double labeling with VGluT1 and other bipolar cell terminal markers. In contrast, P-Rex2 was not expressed in horizontal or amacrine cell processes or terminals or in the dendrites of bipolar or ganglion cells, as indicated by the absence of colocalization with an array of cell- and synapse-specific markers, including calbindin, synaptotagmin 2, PKC, GAD-65, GlyT1, and MAP-1 [21,23,25,28,31,32,34]. Thus, P-Rex2 in the retina is expressed specifically in the specialized glutamatergic ribbon synaptic terminals of photoreceptors and bipolar cells that transmit information vertically through the retina, and not the conventional synapses of amacrine cells or the processes of horizontal cells that mediate lateral processing in the IPL and OPL, respectively.

P-Rex2 localized to both rod and cone photoreceptor terminals in the OPL and to both rod and cone bipolar cell terminals in the IPL, indicating that P-Rex2 expression is not restricted specifically to either rod- or cone-driven synaptic pathways. Similarly, P-Rex2 expression is not exclusively associated with either ON or OFF pathways in the retina as P-Rex2 localized to bipolar cell terminals distributed across the ON and OFF sublayers of the IPL. The distribution of P-Rex2-positive bipolar cell terminals throughout the depth of the IPL further indicates that multiple bipolar cell types contained P-Rex 2 in their terminals. Double labeling for P-Rex2 in conjunction with PKC and synaptotagmin 2 positively identified P-Rex2 expression in the terminals of rod bipolar cells and Type 2 and Type 6 cone bipolar cells [21,28,31]. These studies also showed that the terminals of many additional bipolar cells also contained P-Rex2. On the basis of depth of stratification and the size characteristics of these terminals, it is clear that P-Rex2 also must be present in the terminals of several additional cone bipolar cell types and potentially could be present in the terminals of all bipolar cell types.

The distribution of P-Rex2 does not precisely match the reported distribution of its effector, Rac1. Rac 1 has been localized to photoreceptor outer segments and undergoes light-induced activation [39]. Activation of Rac1 in the outer segment appears to have a key role in light-induced photoreceptor cell death [40,41]. P-Rex2 seems unlikely to modulate light induced activation of Rac1 in the outer segment, as our data show little evidence for enrichment of P-Rex2 in photoreceptor outer segments. In contrast, our data suggest that P-Rex2 may be more important in the regulation of Rac1 activity specifically in photoreceptor terminals. Consistent with this notion, Rac1 has been localized to the distal portion of the mouse OPL [40]. Although Rac1 is known to be important for proper development and polarization of *Drosophila* photoreceptors [42], conditional knockout of Rac1 from mouse rods does not appear to greatly disrupt the structure or function of mouse rods [41], although the structural organization and plasticity of photoreceptor terminals in vertebrate photoreceptors lacking Rac1 has not been examined in detail. The expression of Rac1 by cells in the inner retina and Rac1 labeling in the IPL has been reported previously [40,43], but little is known regarding the cell-specific distribution or activation of Rac1 in the inner retina. The finding that P-Rex2 is selectively localized to bipolar cell terminals suggests that P-Rex2 provides specific regulation of Rac1 activity in those terminals.

The P-Rexes regulate actin cytoskeleton remodeling by activating Rac GTPases. P-Rex activation requires coincident signals via PI3K and G-protein receptor activation [18,19,44] and is a key mechanism for the regulation of membrane dynamics and remodeling of cytoskeleton in response to external cues [11,15,18,19,44,45]. Diminished P-Rex function in neurons leads to aberrations in growth cone structure, membrane ruffling, neurite outgrowth, and neuritic architecture, resulting in functional deficits and impaired synaptic plasticity [11-13,20]. It is likely that P-Rex2 serves a similar function in the terminals of photoreceptors and bipolar cells. One attractive possibility is that P-Rex2 may mediate adaptive remodeling of the terminal in response to simultaneous activation of G-protein and PI3K mediated pathways in the terminal. The terminals of photoreceptors and bipolar cells and their synaptic partners undergo significant anatomical remodeling in response to changes in illumination, including the extension and retraction of processes from the terminal itself and rearrangements associated with post-synaptic processes [46-54]. Plasticity of this nature is best known in the retinas of non-mammalian species [48-53], but adaptive structural changes also occur in mammalian photoreceptor and bipolar cell terminals [46,47,54]. This structural remodeling is dependent at least in part on the actin cytoskeleton as treatment with cytochalaisin D inhibits remodeling [50,52], which would be consistent with a role for P-Rex2 in adaptive remodeling.

Another potential function for P-Rex2 is coordination of adaptive remodeling of the synaptic machinery within photoreceptor and bipolar cell terminals, presumably via activation of Rac1 which is known to be present in photoreceptors and other retinal cells [39-41,43,55]. For example, synaptic ribbons, and active zones in rod photoreceptor terminals undergo adaptive light-dependent (i.e., activity-dependent) remodeling [56-58]. Ribbon and active zone material is removed in the first few hours after light onset resulting in shortening or disappearance of some synaptic ribbons and active zones, and detachment of other ribbons from the terminal plasma membrane. This remodeling is then reversed in darkness. Synaptic vesicle density can also change with light- or dark-adaptation [59]. The mechanism(s) mediating the movement and remodeling of ribbon and active zone material is currently unknown, but P-Rex2-mediated activation of Rac1 leading to local remodeling of actin within the terminal is a plausible contributor.

P-Rex2 also potentially might modulate functional plasticity at photoreceptor and bipolar cell terminals via the regulation of receptors at the surface of the terminal. Double knockout of P-Rex1 and 2 interferes specifically with late phase consolidation of long-term potentiation at the parallel fiber to Purkinje cell synapse in the cerebellum, most likely due to the failure of the synapse to consolidate changes in AMPA receptor density in the absence of P-Rex [20]. It is unlikely that P-Rex2 would modulate functional plasticity at photoreceptor or bipolar cell terminals by direct modulation of transmitter release, as synaptic vesicle exocytosis is not actin-dependent. However, P-Rex2 activation potentially could affect recycling and trafficking of synaptic vesicles in the reserve pool, which are tethered to the actin cytoskeleton by the synaptic vesicle protein synapsin in conventional synapses [60-62]. However, it is not clear whether P-Rex2 might modulate synaptic vesicle interactions with the actin cytoskeleton in photoreceptor and bipolar terminals, which lack synapsins [63].

While the signals that regulate P-Rex2 activity in the terminals of photoreceptors and bipolar cells are not known, P-Rex2 activation requires coincident signaling via PI3K and G-protein-coupled receptor mechanisms [18,19,44]. Furthermore, the available evidence suggests that activated P-Rexes translocate to spatially restricted domains of the plasma membrane in order to activate Rac GTPases [19,45]. Thus, P-Rex2 activation would appear to be ideally suited to tight spatial and temporal regulation of Rac GTPase activation in photoreceptor and bipolar cell terminals in response to very specific combinations of external signals. Although the precise signals that activate P-Rex2 in photoreceptor and bipolar cell terminals are not known, defects in phosphoinositide signaling disrupt the structure, maintenance and function of photoreceptor terminals [64,65] and photoreceptors and bipolar cells possess a variety of G-protein coupled receptors that might contribute to activation of P-Rex2. A challenge for the future will be to define the precise functional role of P-Rex2 in ribbon synaptic terminals and the signals regulating its function.

## Conclusions

These studies indicate that P-Rex2 is associated specifically with the ribbon synaptic terminals of photoreceptors and bipolar cells in the retina. These synapses are highly specialized for rapid glutamate release and serve to transmit visual signals vertically through the retina. The selective expression of P-Rex2 at ribbon synapses suggests a role for P-Rex2 associated specifically with ribbon synapse function that is not shared with the conventional synapses of the retina, which typically utilize inhibitory neurotransmitters and do not show labeling for P-Rex2. Because P-Rex2 is a Rac-GEF, it is likely that it regulates the activity of Rac-GTPases and the actin cytoskeleton in glutamatergic ribbon synaptic terminals of retinal photoreceptors and bipolar cells. It is highly unlikely that actin is directly involved in glutamate release from ribbon synapses, but P-Rex2 would be ideally positioned to modulate rearrangements of the actin cytoskeleton that must accompany plastic changes in the shape and conformation of photoreceptor and bipolar terminals associated with light and dark adaptation.

## Abbreviations

CNS: Central nervous system; GAP: GTPase activating protein; GAD-65: Glutamic acid decarboxylase, 65 kDa isoform; GCL: Ganglion Cell Layer; GEF: Guanine nucleotide exchange factor; GlyT1: Glycine transporter 1; GPCR: G-Protein coupled receptor; INL: Inner nuclear layer; IPL: Inner plexiform layer; MAP-1: Microtubule-associated protein 1; ONL: Outer nuclear layer; OPL: Outer plexiform layer; PNA: Peanut agglutinin; PI3K: Phosphoinositide-3-kinase; PKC: Protein Kinase Cα; P-Rex2: Phosphatidylinositol (3,4,5)-trisphosphate-dependent Rac Exchanger 2; VGluT1: Vesicular glutamate transporter 1.

## Competing interests

The authors declare that they have no competing interests.

## Authors' contributions

DMS performed immunolabeling, microscopy, data analysis, and drafted the manuscript. BAB performed immunolabeling, microscopy, data analysis, and helped to edit the manuscript. Both authors read and approved the final manuscript.
